# Cyclodextrins: Assessing the Impact of Cavity Size, Occupancy, and Substitutions on Cytotoxicity and Cholesterol Homeostasis

**DOI:** 10.3390/molecules23051228

**Published:** 2018-05-20

**Authors:** Lajos Szente, Ashutosh Singhal, Andras Domokos, Byeongwoon Song

**Affiliations:** 1 Cyclolab Cyclodextrin Research and Development Laboratory Ltd., H-1097 Budapest, Hungary; szente@cyclolab.hu; 2 Department of Microbiology, Immunology, and Physiology, Meharry Medical College, Nashville, TN 37208, USA; asinghal@mmc.edu; 3 Center for AIDS Health Disparities Research, Meharry Medical College, Nashville, TN 37208, USA; 4 Department of Chemistry, University of California-Davis, Davis, CA 95616, USA; adomokos@ucdavis.edu

**Keywords:** cholesterol, cyclodextrin, lipids, Niemann–Pick disease type C, occupancy

## Abstract

Cyclodextrins (CDs) are cyclic oligosaccharides; the most common CDs contain six, seven, or eight glucose units called α-CDs, β-CDs, and γ-CDs, respectively. The use of CDs in biomedical research is increasing due to their ability to interact with membrane lipids as well as a wide variety of poorly water-soluble molecules. We assessed the impact of CD cavity size, occupancy, and substitutions on cytotoxicity and cholesterol homeostasis. The potency of CD-mediated cytotoxicity was in the order of β-CDs, α-CDs, and γ-CDs. Substitutions with hydroxypropyl or carboxymethyl group attenuated cytotoxicity compared with the native CDs, whereas CDs substituted with methyl groups exhibited cytotoxicity that was similar to that of the native CDs. The lipid components in blood exerted remarkable hemolysis-alleviating effects in methyl-β-CD-induced hemolysis. Occupancy of the CD cavity with cholesterol or a structurally related lipid molecule abrogated the cytotoxic capacity of the CDs. Interestingly, hydroxypropyl-γ-CD (HPγCD) was able to reduce intracellular cholesterol accumulation in Niemann–Pick disease type C (NPC) patient-derived fibroblasts as efficiently as HPβCD. Proteomic study indicated that HPβCD and HPγCD treatments altered the expression pattern of cellular proteins, suggesting that some of the CD-induced cellular proteins may play an important function in modulating intracellular cholesterol homeostasis.

## 1. Introduction

Cyclodextrins (CDs) are cyclic oligosaccharides that have been generated from starch by enzymatic cleavage of the amylose helix; the three most studied species consist of six, seven, or eight glucopyranose units called α-CDs, β-CDs, and γ-CDs, respectively [1]. CDs present a unique structure with a hydrophobic internal cavity and a hydrophilic outer surface, and can form water-soluble inclusion complexes with a variety of poorly soluble compounds. Due to this property, CDs have been frequently used as drug carriers or in polymeric drug delivery systems for a number of therapeutic applications [1]. We recently demonstrated that sulfobutyl ether β-CD, poly-l-lysine, and hyaluronic acid can form a supramolecular assembly that can encapsulate HIV-1 reverse transcriptase inhibitors such as zidovudine and lamivudine; the drug-loaded supramolecular assemblies exhibited a potent antiviral activity and maintained a sustained drug release in vitro [2]. CDs are also used as an active pharmaceutical agent. Sugammadex, a derivative of γ-CD, is an agent for the reversal of a neuromuscular blockade induced by anesthesia drugs [3]. Hydroxypropyl-β-CD is known to relieve intracellular cholesterol accumulation, and was used in a clinical trial as a potential therapy for treating the Niemann–Pick disease type C (NPC) [4]. A recent study by Alonso et al. has demonstrated that the addition of β-CD and Lactobacillus acidophilus cultures to diet can reduce the levels of total cholesterol and LDL-cholesterol in the blood serum of pigs [5]. The application of CDs in food products and processing is also increasing, and β-CD has been shown to be effective in removing cholesterol from milk [6,7,8].

CDs, in particular the β-CDs, are able to interact with cell membranes and are known to extract cholesterol and other lipids from the membranes [9,10,11]. This membrane-disrupting activity can alter the integrity and functions of certain membrane domains, depending on the type and concentration of the CD. The cholesterol extraction capacity of β-CDs is greater than that of γ-CDs or α-CDs [9,12]. Due to their membrane interaction activity, the methylated β-CDs have been routinely used as a cell biology tool for manipulating membrane structure and function [13,14,15,16]. Methylated β-CDs are also the most powerful drug-solubilizing agents amongst all of the known cyclodextrins [17]. Despite their potential as a drug carrier, methylated β-CDs can induce hemolysis in a concentration-dependent manner through the solubilization and sequestration of membrane lipids, especially cholesterol [12]. Due to their hemolytic effect, methylated β-CDs are rarely considered parenteral drug carriers. However, most of the available data on the hemolytic effect of methylated β-CDs have been obtained from in vitro experiments made on isolated and washed erythrocytes without considering the blood plasma components. The hemolytic EC_50_ values of methylated β-CDs were found to be 4.8 mM and 23.8 mM in saline media and blood plasma, respectively [18], suggesting that the conditions of in vitro hemolysis tests influence the hemolytic potency of the CDs. The cytotoxic (e.g., hemolytic) capacities of CDs [9,19] need to be carefully evaluated for safe use in pharmaceutical applications.

NPC disease is an autosomal recessive lipid storage disorder that is characterized by progressive neurodegeneration and a massive accumulation of free cholesterol in the late endosomes and lysosomes [20]. Upon entry into the cell via endocytosis through the LDL receptor, cholesterol is transported into early endosomes, late endosomes, and lysosomes, and cholesterol is then exported from the late endosomes/lysosomes (LE/LY) via the concerted action of the lysosomal proteins NPC1 [21] and NPC2 [22]. Approximately 95% of NPC cases are caused by mutations in the *NPC1* gene, while 5% of the cases are caused by mutations in the *NPC2* gene. Clinical manifestations of NPC disease include severe neuronal degeneration and hepatosplenomegaly [20]. In one model of NPC1/2 function, lysosomal acid lipase (LAL) hydrolyzes LDL-associated cholesteryl ester; the free cholesterol is first transferred from LAL to NPC2, then from NPC2 to the N terminal domain of NPC1, and is then exported out of the lysosomal compartment by poorly understood mechanisms [20].

Hydroxypropyl-β-CD (HPβCD) is known to have efficacy for NPC disease and rescue the cholesterol accumulation defect in human cells with *NPC1* or *NPC2* mutations [20,23,24,25]. Recently, hydroxypropyl-γ-CD (HPγCD) was also shown to relieve the cholesterol accumulation defect in a mouse model of NPC disease [26]. The mechanisms of HPβCD and HPγCD for modulating intracellular cholesterol accumulation need to be defined, considering that the physicochemical properties of HPγCD are different from those of HPβCD [1] and the cholesterol extraction potency of HPγCD is much weaker than that of HPβCD [10]. The purpose of this study is to determine the mechanisms of CD-induced cytotoxicity and evaluate the abilities of CD derivatives for modulating intracellular cholesterol homeostasis. Understanding how the CDs relieve cholesterol accumulation in *NPC* mutant cells will contribute to the development of effective therapeutic approaches for neurodegenerative lipid storage disorders such as NPC. In the present study, we evaluated 12 CD derivatives for their cytotoxic activity, defined the mechanisms of methyl-β-CD-induced cytotoxicity, and evaluated CD derivatives for their cholesterol-lowering effect in NPC patient-derived fibroblasts.

## 2. Results and Discussion

### 2.1. Cytotoxic Activity of CDs in Human Cell Lines

**Figure 1:**
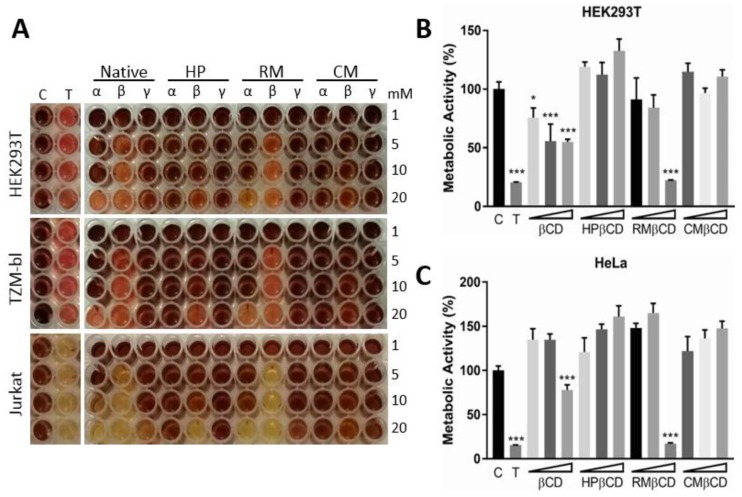
Effect of cyclodextrin (CD) derivatives on cell viability in human cell lines HEK293T, TZM-bl, Jurkat, and HeLa. CDs tested in this experiment include the native forms as well as their substitutions with hydroxypropyl (HP), randomly methylated (RM), or carboxymethyl (CM) groups: CDs were added at 1 mM, 5 mM, 10 mM, and 20 mM for experiments in (**A**) and 1 mM, 3.3 mM, and 10 mM for experiments in (**B**) and (**C**). Cells treated with phosphate buffer saline (PBS) and Triton X-100 were included as negative and positive controls for cytotoxicity, respectively: C, PBS treatment; T, Triton X-100 treatment. The cell viability (metabolic activity) was determined using the CellTiter 96 AQueous One Solution Cell Proliferation Assay Kit (Promega, Madison, WI, USA). Data are represented as means ± S.D. (*n* = 3). * *p* < 0.02 vs. control; *** *p* < 0.001 vs. control.

We tested the effect of 12 CD derivatives on cell viability and cytotoxicity in vitro in human embryo kidney-derived HEK293T cells, human cervical cancer-derived HeLa and TZM-bl cells, and human T-lymphocyte-derived Jurkat cells. The CD derivatives that were used in this study include α-CDs, β-CDs, and γ-CDs as the native forms, and as their derivatives such as hydroxypropyl (HP), randomly methylated (RM), or carboxymethyl (CM) forms. Cells were treated with each of the CD derivatives at concentrations ranging from 1 mM to 20 mM for 24 h, and then subjected to an MTS-based cell viability assay using the CellTiter 96 AQueous One Solution Cell Proliferation Assay Kit (Promega, Madison, WI, USA), and a cytotoxicity assay using the LDH Cytotoxicity Assay Kit (Thermo Fisher Scientific, Waltham, MA, USA). Triton X-100 (TX) was included as a positive control for cytotoxicity. Cell viability (or metabolic activity) was not affected by CDs at 1 mM, but it was reduced by some CDs at or above 5 mM, as shown by detection of a colored formazan product (Figure 1A) and by measurement of absorbance at 490 nm (Figure 1B,C); the order of the CD-induced impact on cell viability was β-CD > α-CD > γ-CD. Interestingly, substitution with hydroxypropyl or carboxymethyl groups attenuated the CD-induced impact on cell viability compared with the native CDs, whereas randomly methylated CDs showed an effect similar to or slightly higher than the native CDs. These findings were confirmed by the lactate dehydrogenase (LDH) assay (data not shown), which measures the levels of the cytosolic enzyme lactate dehydrogenase that had been released into the culture medium, which is indicative of cell death.

There are potentially hundreds or thousands of variations of CDs that have different ring sizes and random or site-specific chemical substitutions. It is believed that the molecular dimensions of the β-CD cavity (diameter 0.60–0.65 nm and height 0.78 nm) make it the most ideal host among the three native CDs for inclusion complex formation with the most drugs, flavors, cosmetic ingredients, and pesticides [1,27]. To determine whether there is a correlation between the cytotoxic capacity and the cholesterol solubilizing ability, we measured the cholesterol-solubilizing activity of CD derivatives (Table 1). Methyl-β-CDs (DIMEB-50, DIMEB-95, and RAMEB) showed much higher cholesterol-solubilizing activity compared with HPβCD or sulfobutyl ether β-cyclodextrin (SBEβCD), whereas HPγCD was not able to solubilize cholesterol. These results are consistent with the notion that β-CD has an affinity for cholesterol [28,29], and it can extract cholesterol and other membrane lipid components from cells [30]. Overall, our findings suggest that the cavity size of β-CD is more effective for CD binding to the membrane cholesterol compared with that of γ-CD, and the substitution groups of CDs influence their ability to bind membrane cholesterol and evoke cell damage. For example, the methyl groups on β-CD allow higher cytotoxic ability, whereas substitutions with hydroxypropyl or sulfobutyl ether groups significantly attenuate the cytotoxic ability of β-CD.

### 2.2. Hemolytic Activity of Methyl-β-CDs In Vitro

Based on the significant cytotoxic effect of methyl-β-CDs in cell culture, we determined the hemolytic capacity of methyl-β-CDs at 10 to 14 different CD concentrations on isolated human erythrocytes in the absence or presence of blood plasma constituents. The data shown in Table 2 list the EC_50_ values of different β-CD derivatives on human erythrocytes when the test was performed with isolated human erythrocytes in phosphate buffer saline (PBS) solution as well as in PBS solution containing different amounts of blood plasma. In the absence of blood plasma, the in vitro hemolysis-provoking potency of methyl-β-CDs was found to decrease as follows: DIMEB (50 and 95) > randomly methylated β-cyclodextrin (RAMEB) > heptakis (2,3,6-tri-*O*-methyl)-β-cyclodextrin (TRIMEB). The addition of blood plasma caused an increase in the EC_50_ values of CD derivatives, with its addition having a larger impact on heptakis (2,6-di-*O*-methyl)-β-cyclodextrin (DIMEB)-mediated hemolysis compared with hemolysis mediated by RAMEB or TRIMEB. The hemolytic EC_50_ values of DIMEB-50 and DIMEB-95 were practically identical in the traditional in vitro hemolysis tests, whereas in the presence of plasma, DIMEB-50 was less hemolytic than DIMEB-95. This refers to a correlation between the isomeric purity and hemolytic activity of methyl-β-CD products. The higher the isomeric purity, the more hemolytic the DIMEB.

**Figure 2:**
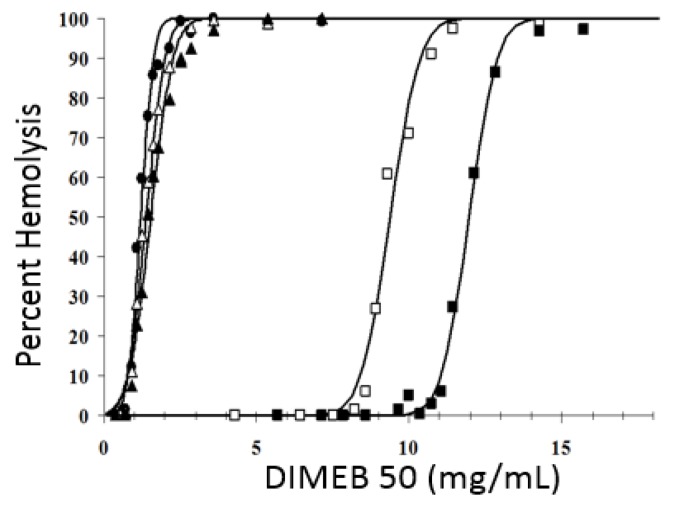
Effect of bovine serum albumin and human blood plasma on the hemolytic potency of DIMEB-50: (●) PBS, EC_50DIMEB_ = 1.2 mg/mL; (△) 5 mg/mL BSA, EC_50DIMEB_ = 1.38 mg/L; (▲) 10 mg/mL bovine serum albumin (BSA), EC_50DIMEB_ = 1.53 mg/mL; (☐) 100-µL plasma resulting in 5 mg/mL protein concentration in the mixture, EC_50DIMEB_ = 9.38 mg/mL; (■) 200-µL plasma resulting in 10 mg/mL protein concentration in the mixture, EC_50DIMEB_ = 11.96 mg/mL.

To demonstrate the specificity of the anti-hemolytic activity mediated by blood plasma, the hemolytic assay was conducted in the presence of human plasma or bovine serum albumin (BSA). The addition of human plasma caused an increase in the EC_50_ values in a dose-dependent manner, whereas the addition of BSA did not alleviate the cell damage caused by DIMEB (Figure 2). These results provide experimental evidence for the role of plasma components in protecting cells against CD-induced hemolytic activity. Overall, these results suggest a significant protective effect of blood plasma components against CD-induced cytotoxicity.

### 2.3. Impact of Blood Plasma Lipids on the Hemolytic Activity of Methyl-β-CDs

It is possible that CD-induced hemolysis might be caused by entrapment of the membrane lipid components by the CD. We hypothesized that blood plasma lipids/lipoproteins could compete with the membrane-associated lipids to form inclusion complexes with the methyl-β-CDs. This may be one of the mechanisms by which blood plasma reduces the active hemolytic concentration of the CD derivatives [31]. To test this hypothesis, we conducted a set of experiments to determine the hemolytic effect of DIMEB-50 in washed red blood cells (RBCs), in washed RBCs supplemented with plasma, and in whole blood samples containing different lipid contents. As shown in Table 3, a rather clear relationship exists between the actual cholesterol/triglyceride levels and the overall protective effect of plasma or whole blood against CD-induced hemolysis. The results of these experiments suggest that the necessary concentrations of dimethyl-β-cyclodextrin (DIMEB-50) to cause the same extent of hemolysis are directly proportional to the actual lipid (cholesterol and triglyceride) levels of the blood samples. However, there was no significant difference in the hemolysis sensitivity of isolated red blood cells taken from persons with normal versus elevated plasma cholesterol levels. The sensitivity of isolated erythrocytes against CD-provoked hemolysis appeared the same regardless of their previous cholesterol or lipid environment, but the hemolytic sensitivity changed according to the levels of cholesterol/triglyceride present in the blood that is added to the hemolysis test.

This study demonstrated a significant difference in the hemolysis-provoking effect of methyl-β-CDs when isolated red blood cells and whole blood were used as test media. The lipid components in the blood plasma were found to alleviate the hemolytic activity caused by methyl-β-CDs. The results presented in this study are in agreement with the findings by Kiss et al. that demonstrated a correlation between the cytotoxic effect, hemolytic activity, and the cholesterol complexation properties of CD derivatives [32]. These observations suggest that the results of routine in vitro hemolysis tests on isolated red blood cells cannot be simply translated into in vivo (whole blood) conditions. The parenteral toxicity of methyl-β-CDs is obviously not negligible, but the reported in vitro hemolytic EC_50_ values need to be reconsidered in the light of the results presented here. The parenteral application of low doses (around 10 mg) of DIMEB as a potent drug solubilizer or carrier is still promising.

### 2.4. Abrogation of Cytotoxic Activity by Cholesterol Occupancy in the CD Cavity

**Figure 3:**
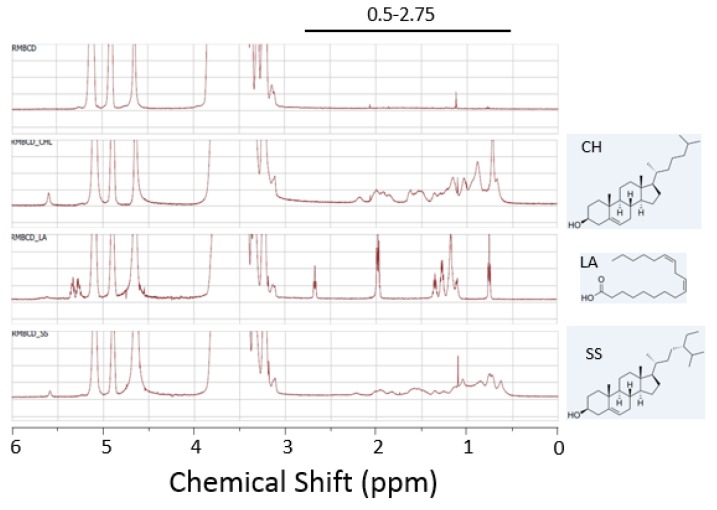
NMR spectra of the CD/lipid complexes. ^1^H-NMR spectra of the free cyclodextrin RAMEB (top) and the RAMEB complexes with cholesterol (CH), linoleic acid (LA), and β-sitosterol (SS) are shown. Chemical structures of the lipid guest molecules are shown next to the spectra. The peaks unique to the lipid guest molecules were detected at 0.5–2.75 ppm.

We hypothesized that the cavity occupancy or the ability to form inclusion complexation is critical for the capacity of CDs to evoke cell damage through the extraction of the membrane lipids. To this end, we generated randomly methylated β-CD complexed with cholesterol, β-sitosterol, or linoleic acid (Figure 3). We confirmed complexation of the CD with the lipid molecules using NMR spectroscopy (Figure 3). ^1^H-NMR spectroscopy of the CD/lipid complexes indicated the presence of peaks unique to the lipid guest molecules (cholesterol, linoleic acid, and β-sitosterol) at 0.5–2.75 ppm (Figure 3). These results together suggested that methyl-β-CD formed inclusion complexes with the lipid guest molecules (cholesterol, linoleic acid, and β-sitosterol) within the CD cavity.

**Figure 4:**
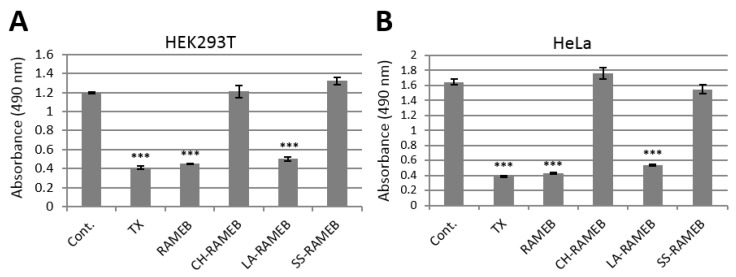
Cytotoxic effect of the lipid-loaded methyl-β-cyclodextrin complexes (10 mM) in HEK293T cells (**A**) and HeLa cells (**B**). Cont., PBS; TX, Triton X-100; RAMEB, randomly methylated β-CD; CH-RAMEB, cholesterol-loaded RAMEB; LA-RAMEB, linoleic acid-loaded RAMEB; SS-RAMEB, β-sitosterol-loaded RAMEB. Cell viability was determined using the CellTiter 96 Aqueous One Solution Cell Proliferation Assay Kit (Promega). Data are represented as means ± S.D. (*n* = 3). *** *p* < 0.001 vs. control.

The CD/lipid inclusion complexes were then tested for their ability to reduce the metabolic activity (or cell viability) in HEK293T cells and HeLa cells (Figure 4) using the CellTiter 96 AQueous One Solution Cell Proliferation Assay Kit (Promega). Cells were treated with 10 mM of the free RAMEB, cholesterol-loaded RAMEB, linoleic acid-loaded RAMEB, or β-sitosterol-loaded RAMEB. At 24 h after treatment, a cell viability assay was conducted, and the absorbance at 490 nm was measured to determine the levels of the reaction products that were generated in metabolically active cells. Triton X-100 (TX) was included as a positive control for cytotoxicity. As expected, the free RAMEB caused a significant reduction in metabolic activity in both HEK293T cells (Figure 4A) and HeLa cells (Figure 4B), which was similar to the Triton X-100 control. Interestingly, treatment with the cholesterol-loaded or β-sitosterol-loaded RAMEB did not affect cell viability, whereas treatment with the linoleic acid-loaded RAMEB reduced cell viability similar to the free RAMEB. Based on the chemical structures of the lipid molecules (Figure 3), these results suggest that the association constants of cholesterol and β-sitosterol for binding to RAMEB are relatively high, whereas the association constant of linoleic acid for binding to RAMEB is very low. The RAMEB/linoleic acid complex will therefore dissociate easily, and the free RAMEB will be available for interacting with and extracting the membrane lipid components such as cholesterol, resulting in cell damage. Overall, these results suggest that the CD cavity must be empty to cause membrane damage and cell death.

### 2.5. Effects of CDs on Intracellular Cholesterol Distribution

**Figure 5:**
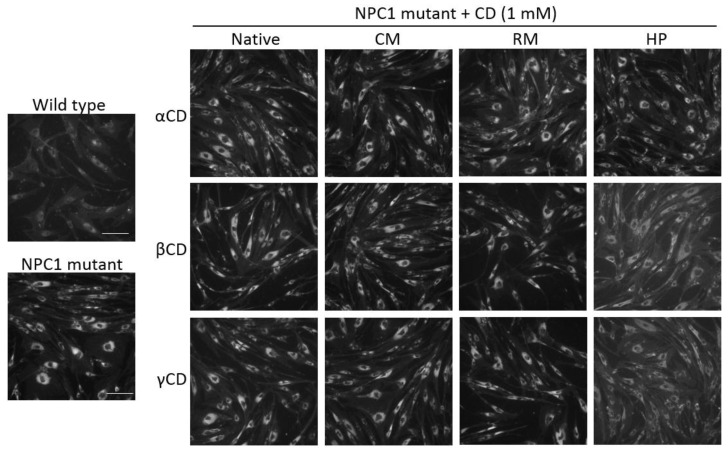
Effect of CD derivatives on the intracellular levels of free form of cholesterol. Primary fibroblast cells from a healthy donor or Niemann–Pick disease type C (NPC) patient were incubated with CD derivatives (1 mM) for 72 h, and the levels of free cholesterol were determined by staining with Filipin III. Data shown are representative of three independent experiments. Wild type, primary fibroblast cells from a healthy donor; *NPC1* mutant, primary fibroblast cells from an NPC patient. The size of a scale bar is 200 μm.

We determined the effects of the CD derivatives on intracellular cholesterol accumulation in primary fibroblast cells derived from an NPC patient using Filipin III, a fluorescent compound that specifically binds to unesterified cholesterol. After treatment with the CD derivatives (1 mM) for 72 h, the cells were examined for the fluorescent intensity of Filipin III using a fluorescence microscope. At 1 mM, the CDs did not cause any cytotoxic effects in human cell lines (Figure 1) and primary fibroblasts (data not shown). The primary fibroblast cells of the NPC patient showed much higher levels of intracellular cholesterol, reflecting a defect in cholesterol trafficking, compared with the primary fibroblast cells of a healthy donor control (Figure 5, left). Of the 12 CD derivatives tested, including the native forms or HP, RM, or CM-functionalized forms of α-CDs, β-CDs, and γ-CDs, HPβCD and HPγCD caused a reduction in cholesterol accumulation in *NPC1* mutant cells, whereas other CD derivatives did not relieve the cholesterol accumulation defect (Figure 5). Our findings are in agreement with previous studies demonstrating the effects of HPβCD and HPγCD in the modulation of cholesterol accumulation and the alleviation of NPC disease [20,23,24,25,26]. Studies are underway to define the molecular mechanisms by which HPβCD and HPγCD alleviate the cholesterol-trafficking defect.

### 2.6. Effects of HPβCD and HPγCD Treatments on the Expression of Cellular Proteins

**Figure 6:**
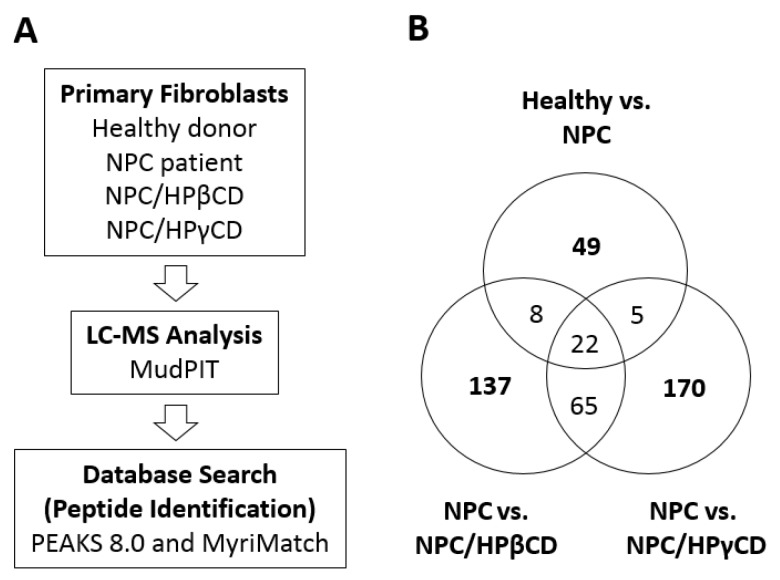
LC-MS proteomic analysis of NPC patient-derived fibroblasts that were treated with HPβCD or HPγCD. (**A**) NPC patient-derived fibroblasts were treated with HPβCD or HPγCD (1 mM, 72 h) and cell extracts were subjected to LC-MS analysis (MudPIT) and peptide identification using PEAKS8.0 and MyriMatch. Untreated fibroblasts from an NPC patient and untreated fibroblasts from a healthy donor were included as controls. Four groups of samples were analyzed; untreated healthy donor cells (Healthy), untreated NPC patient cells (NPC), NPC patient cells treated with HPβCD (NPC/HPβCD), and NPC patient cells treated with HPγCD (NPC/HPγCD); (**B**) Identification of proteins whose levels differed more than two-fold in pairwise comparison; 49 proteins in healthy vs. NPC, 137 proteins in NPC vs. NPC/HPβCD, and 170 proteins in NPC vs. NPC/HPγCD.

To understand the potential mechanisms of the CDs for modulating intracellular cholesterol homeostasis, we tested the possibility that HPβCD and HPγCD treatments may alter the expression profile of cellular proteins. NPC patient-derived fibroblasts (*NPC1* mutant) were treated with HPβCD or HPγCD (1 mM, 72 h) and subjected to trypsin digestion and LC-MS/MudPIT analysis (Figure 6A). Untreated NPC patient-derived cells and untreated healthy donor cells were included as controls. Database searches and peptide identification were conducted using PEAKS (version 8.0, Bioinformatics Solutions Inc., Waterloo, ON, Canada) and MyriMatch (version 2.0, OMICTOOLS, Le-Petit-Quevilly, France). Pairwise comparisons were made using the identified proteins from each experimental group: healthy versus NPC, NPC versus NPC/HPβCD, and NPC versus NPC/HPγCD (Figure 6B). When untreated fibroblasts of a healthy donor and NPC patient were compared, there were 49 proteins whose levels differed more than two-fold. When NPC patient-derived fibroblasts (*NPC1*^−/−^) were treated with HPβCD or HPγCD, there were 137 and 170 proteins, respectively, whose levels were altered more than two-fold by the CD treatment.

The 49 proteins detected in the healthy versus NPC comparison may be implicated in causing the cellular abnormality such as cholesterol accumulation in *NPC1* mutant cells. Interestingly, 35 proteins out of the 49 potential NPC disease-specific proteins were altered more than two-fold upon treatment with HPβCD or HPγCD; their levels were shifted toward those of healthy donor cells from those of NPC patient-derived cells (manuscript in preparation). Studies are in progress to determine the roles of CD-induced cellular proteins in the regulation of cellular cholesterol homeostasis.

### 2.7. Mechanisms of CDs for Modulating Intracellular Cholesterol Accumulation

**Scheme 1:**
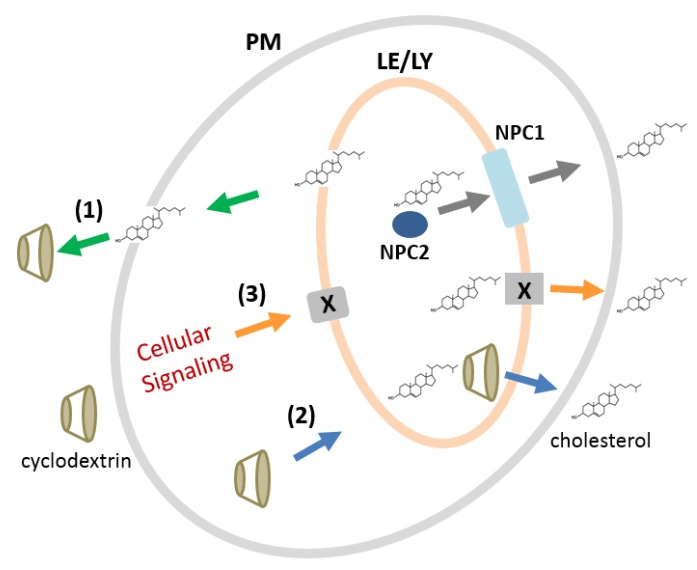
Potential mechanisms by which CDs can rescue the cholesterol accumulation defect in an NPC disease model. In healthy donor-derived cells, the NPC1 and NPC2 proteins execute the export of cholesterol from the late endosomes/lysosomes (grey arrows). In NPC patient-derived cells with NPC1 or NPC2 deficiency, cholesterol accumulates within the late endosomes/lysosomes. The CDs may rescue the cholesterol accumulation defect by extracting cholesterol at the plasma membrane followed by the movement of intracellular cholesterol (mechanism 1: green arrows), by entering the cells by endocytosis and directly binding and exporting cholesterol (mechanism 2: blue arrows), or by inducing cellular signaling pathways at the plasma membrane or following endocytosis (mechanism 3: orange arrows). The protein “X” indicates the CD-induced cellular protein(s) that may play a role in cholesterol trafficking in mechanism 3. PM, plasma membrane; LE/LY, late endosome/lysosome; NPC1, Niemann–Pick disease type C1 protein; NPC2, Niemann–Pick disease type C2 protein.

The mechanisms of CDs for modulating intracellular cholesterol homeostasis are not clear. There are several mechanisms that can explain the roles of CDs in cholesterol homeostasis in an NPC disease model (Scheme 1). First, CDs can extract cholesterol from the plasma membrane, and then, cholesterol from the intracellular compartments moves to the plasma membrane to replace the lost cholesterol. Second, CDs can enter the cell via endocytosis and directly bind and export cholesterol from the endolysosomal compartments (late endosomes and lysosomes). Third, CDs, either at the plasma membrane or after endocytosis, can induce cellular signaling pathways that promote intracellular cholesterol trafficking. We demonstrated that HPγCD can rescue the cholesterol accumulation defect (Figure 5), whereas it has no ability to solubilize cholesterol (Table 1). These findings favor the third mechanism (“cellular signaling”) as a potential mechanism for the CDs, in particular HPγCD, in the regulation of cholesterol homeostasis, although we cannot exclude the first and second mechanisms.

Overall, our findings in the present study suggest that CD derivatives may have a multitude of mechanisms for modulating cellular cholesterol homeostasis by interacting with the membrane lipid/cholesterol and/or inducing cellular signaling pathways, whereas the cholesterol binding/solubilizing capacity of CDs, in particular methyl-β-CDs, can dictate membrane damage and cell death depending on the CD concentration. We have evidence that some of the cellular proteins that were upregulated in response to the HPβCD or HPγCD treatments are involved in promoting cholesterol export from the late endosomes/lysosomes (manuscript in preparation). Further studies are warranted in order to understand the detailed mechanisms by which HPβCD and HPγCD rescue the cholesterol-trafficking defect in an NPC disease model. A better understanding of the mechanisms of the CDs in the regulation of cellular cholesterol homeostasis will lead to improved strategies for neurodegenerative lipid storage disorders such as NPC.

## 3. Materials and Methods

### 3.1. Materials

The CD derivatives that were used in this study are products of Cyclolab (Budapest, Hungary). These CDs include α-CDs, β-CDs, and γ-CDs of native forms as well as hydroxypropyl, methyl, and carboxymethyl substitutions. Methyl β-CDs include: heptakis (2,6-di-*O*-methyl)-β-CD, isomeric purity 50% (DIMEB-50); heptakis (2,6-di-*O*-methyl)-β-CD, isomeric purity 95% (DIMEB-95); randomly methylated β-CD with an average degree of methylation of 13 methyl groups per βCD ring (RAMEB); and heptakis (2,3,6-tri-*O*-methyl)-β-CD (TRIMEB). Cell culture medium and reagents were purchased from Thermo Fisher Scientific (Waltham, MA, USA); these include Dulbecco’s modified Eagle’s medium (DMEM), RPMI 1640 medium, fetal bovine serum, penicillin, and streptomycin. A CellTiter 96 Aqueous One Solution Cell Proliferation Assay System was purchased from Promega (Madison, WI, USA). An LDH Cytotoxicity Assay Kit was obtained from Thermo Fisher Scientific (Waltham, MA, USA). Cholesterol, linoleic acid, and β-sitosterol were obtained from Sigma-Aldrich (St. Louis, MO, USA). Deuterated water (D_2_O) used in NMR analysis was purchased from Cambridge Isotope Laboratories (Tewksbury, MA, USA). Filipin III were obtained from Sigma-Aldrich (St. Louis, MO, USA).

### 3.2. Cell Culture

The human embryo kidney cell line HEK293T, human cervical cancer-derived cell line HeLa, and human T-lymphocyte-derived cell line Jurkat were obtained from the American Type Culture Collection (ATCC). The TZM-bl cell line is a HeLa-derived cell line expressing CD4, CXCR4, CCR5, and the HIV LTR-luciferase, and this cell line was obtained from the NIH AIDS Reagent Program (NIH, Bethesda, MD, USA). HEK293T, HeLa, and TZM-bl cells were maintained in DMEM medium supplemented with 10% heat-inactivated fetal bovine serum, 100 U/mL of penicillin, and 100 μg/mL of streptomycin at 37 °C in a 5% CO_2_ humidified incubator. Jurkat cells were maintained in RPMI 1640 medium supplemented with 10% heat-inactivated fetal bovine serum, 100 U/mL of penicillin, and 100 μg/mL of streptomycin at 37 °C in a 5% CO_2_ humidified incubator. Primary fibroblasts from an Niemann–Pick disease type C1 patient (GM03123) and healthy donor (GM05659) were obtained from Coriell Institute (Camden, NJ, USA) and maintained in DMEM medium supplemented with 15% fetal bovine serum (not inactivated), 100 U/mL of penicillin, and 100 μg/mL of streptomycin at 37 °C in a 5% CO_2_ humidified incubator.

### 3.3. Cell Viability and Cytotoxicity Tests

The effects of CD treatment on cell viability and cytotoxicity were determined using the CellTiter 96 AQueous One Solution Cell Proliferation Assay Kit (Promega) and LDH Cytotoxicity Assay Kit (Thermo Fisher Scientific), respectively. Cells were seeded in 96-well plates at a density of 1–3 × 10^4^ cells per well. One day after seeding, cells were treated with a series of dilutions of the CDs. Cells treated with phosphate buffered saline (PBS) and Triton X-100 (0.1%) were included as negative and positive controls, respectively. After 24 h to 48 h incubation at 37 °C, cells were subjected to cell viability and cytotoxicity tests according to the manufacturer’s instructions. The CellTiter 96 AQueous One Solution Cell Proliferation Assay is based on the conversion of the tetrazolium compound [3-(4,5-dimethylthiazol-2-yl)-5-(3-carboxymethoxyphenyl)-2-(4-sulfophenyl)-2H-tetrazolium, inner salt; MTS] into a colored formazan product by dehydrogenase enzymes in metabolically active cells [33], thus providing a convenient colorimetric method for determining the metabolic activity (or cell viability). The absorbance of the plates was recorded at 490 nm with an xMark microplate absorbance spectrophotometer (Bio-Rad, Hercules, CA, USA). The data were normalized to the values for untreated or PBS-treated control. The LDH Cytotoxicity Assay Kit measures the amount of a cytosolic enzyme lactate dehydrogenase (LDH) released into the cell culture medium, thus measuring the membrane damages that occur as the result of cell death. Extracellular LDH in the media can be quantified by a coupled enzymatic reaction in which LDH catalyzes the conversion of lactate to pyruvate via nicotinamide adenine dinucleotide (NAD^+^) reduction to NADH. Diaphorase then uses NADH to reduce a tetrazolium salt (INT) to a red formazan product that can be measured at 490 nm. The level of formazan formation is directly proportional to the amount of LDH released into the medium, which is indicative of cytotoxicity.

### 3.4. Cholesterol Solubilization by CDs

CD derivatives were dissolved in distilled water at a concentration of 50 mM. An excess amount of cholesterol was added into a glass vial containing 2 mL of 50 mM CDs. The vials were sealed and stirred with 600 r.p.m. at room temperature for 24 h protected from light. The aqueous suspension was filtered through a cellulose acetate membrane with 0.4 micrometers pore size. The cholesterol contents of the clear filtrates were determined by HPLC using Nucleosil 120-5 C_18_ 4 mm × 100 mm column (Macherey Nagel, Bethlehem, PA, USA) at 40 °C, UV detection at 210 nm, and acetonitrile:isopropanol (3:1) as eluent. The average of three parallel runs for each was calculated.

### 3.5. In Vitro Hemolysis Tests Using Isolated Human Erythrocytes

Venous blood was taken from healthy volunteers and placed in plastic tubes containing sodium–citrate anti-coagulant. The cell fraction including erythrocytes was separated from the plasma components by centrifugation (2500× *g*, 10 min, 20 °C), washed three times with phosphate buffered saline (PBS) solution, and re-suspended in the same solution. The erythrocyte count was adjusted to 10^9^ per mL under the control of a Laborscale cell analyzer (Medicor, Hungary). The test was performed in standard Eppendorf-type plastic tubes, where the incubation mixture consisted of 1.3 mL of reagent solution containing increasing concentrations of CDs dissolved in PBS, 0.05 mL of erythrocyte suspension containing 5 × 10^7^ red blood cells, and 0.05 mL of 3% Na_2_CO_3_. After 10 min incubation at 37 °C, samples were mixed and centrifuged at 5000x g. The absorbance of the released hemoglobin in the supernatant was determined at 540 nm spectrophotometrically. As a reference, total (100%) hemolysis was obtained by replacing PBS with distilled water. CD-induced hemolysis was related to this reference value and expressed as percent hemolysis. All of the experiments included at least 10 different concentrations of the test materials in triplicate runs to allow the plotting of dose-effect curves from multi-point determinations, and to calculate EC_50_ values. In order to investigate the protective effect of plasma components, aliquots of PBS were replaced by 50 µL, 100 µL, and 200 µL of blood plasma in the incubation mixture. Plasma protein concentration was determined by the biuret method.

### 3.6. In Vitro Hemolysis Test in Whole Blood Samples

For these experiments, healthy volunteers with identical main blood groups [A, Rhesus blood factor D (Rh) positive] but differing in their plasma lipid contents were selected. Plasma and cellular blood components were separated from the freshly taken sodium-citrated blood by centrifugation (2500× *g*, 10 min, 4 °C). The sedimented composite cellular fraction (red and white blood cells, and platelets) was washed with PBS, then re-suspended either in their own original plasma or in the foreign plasma with the identical blood group, resulting in erythrocyte/plasma combinations of normal cholesterol/lipid levels or higher cholesterol/lipid levels. The erythrocyte count was adjusted to 4 × 10^9^ per mL in the blood. In this case, hemolysis test mixtures contained 0.04 mL of test solution with increasing concentrations of methyl-β-CD, 0.25 mL of blood, and 0.01 mL of 3% Na_2_CO_3_. Incubation and subsequent centrifugation conditions were identical to the above. Aliquots of the supernatant were diluted using PBS (containing 3% Na_2_CO_3_), and their hemoglobin content was determined. Again, dose-effect curves were constructed from multi-point determinations of triplicate runs. The levels of cholesterol, high-density lipoprotein (HDL), and triglyceride in plasma were determined by the use of standard laboratory procedures [34].

### 3.7. Generation of Cyclodextrin/Lipid Complexes

Lipid-loaded randomly methylated β-CD (RAMEB) was generated as follows. One gram of RAMEB was dissolved in 10 ml of water while stirring; this becomes a clear solution. To the RAMEB solution, 1 g of lipids (cholesterol, β-sitosterol, or linoleic acid) was added while stirring; this becomes a turbid suspension with some lipid undissolved. After stirring for 24 h at room temperature, the mixture was filtered through a 0.45-micron filter. The filtrate was frozen at −80 °C, and then lyophilized overnight.

### 3.8. NMR Spectroscopy

To prepare samples for NMR spectroscopy, 25 mg or 50 mg of RAMEB or lipid-loaded RAMEB were weighed out, and excess D_2_O was added to the vial containing the sample. The sample was frozen in a −80 °C freezer and then lyophilized overnight to achieve deuterium exchange and minimize the appearance of broad hydroxyl peaks of the CD in the NMR spectra. To the fluffy white powder generated after the lyophilization process, 1 mL of D_2_O was added to create a sample with the concentration of 25 mg/mL or 50 mg/mL. Of this dissolved sample, an aliquot of 600 µL was transferred into a clean NMR tube, and the sample was analyzed using a 600-MHz Varian VNMRS Spectrometer (Agilent Technologies, Santa Clara, CA, USA). The obtained data were then processed using Mnova software (version 11.0.1, Mestrelab, Escondido, CA, USA).

### 3.9. Intracellular Distribution of Cholesterol

Cells were incubated in medium containing CD derivatives at 1 mM for 72 h. Cells were washed three times with PBS and fixed with 3.7% paraformaldehyde for 30 min at room temperature. After washing with PBS, cells were stained with PBS containing Filipin III (12.5 μg/mL) for 30–45 min at room temperature in the dark. After washing with PBS and applying a mounting medium, cells were analyzed using a Nikon TE2000 widefield microscope (Nikon, Melville, NY, USA).

### 3.10. MudPIT Proteomic Analysis

Untreated healthy donor cells, untreated NPC1 mutant cells, or NPC1 mutant cells treated with HPβCD or HPγCD (1 mM for 72 h) were re-suspended with 0.1% Triton X-100 in PBS in the presence of protease inhibitors. Proteins (approx. 50 µg total protein) were denatured in 8 M urea and 50 mM Tris-HCl, pH 8.0, reduced with 10 mM TCEP for 60 min, alkylated with 50 mM iodoacetamide for 60 min, and then diluted with water to 2 M urea and 50 mM Tris-HCl, pH 8.0. Two micrograms of trypsin (Promega) were added for overnight digestion (18 h), and then the tryptic peptides were desalted using Pierce C18 spin columns (Thermo Fischer Scientific). Desalted samples were dried in Speed-Vac, resuspended in 5 μL of 0.5% formic acid (FA), and loaded onto a 3-phase MudPIT column, as described previously [35]. A 10-step MudPIT was executed for LC-MS analysis using an Eksigent™ AS-1 autosampler and an Eksigent™ 1D Plus nano-LC pump online with an Orbitrap LTQ XL linear ion trap mass spectrometer (Thermo Fisher Scientific, Waltham, MA, USA) with a nanospray source. MS data acquisition was done in a data-dependent six-event method [a survey FTMS scan (res. 30,000), followed by five data-dependent IT scans for the five consequent most abundant ions]. Database searches were done with PEAKS (version 8.0, Bioinformatics Solutions Inc., Waterloo, ON, Canada) and MyriMatch (version 2.0, OMICTOOLS, Le-Petit-Quevilly, France) against the forward and reverse human trypsin sequences (as downloaded from GenBank). The parameters for database search were: full tryptic digestion; up to two missed cleavage sites; 10 ppm for peptide mass tolerance; 0.5 Da for fragment mass tolerance; cysteine carbamidomethylation (+57 Da) as fixed modification; methionine oxidation (+16 Da) as variable modification. The relative quantification of the identified proteins was performed with the Q module of the PEAKS software pack based on the extracted ion currents of the identified unique peptides’ parent ions.

### 3.11. Data Analysis

Experiments were performed in triplicate and repeated at least three times. Data are presented as means ± SD.

## 4. Conclusions

This study demonstrated that β-CDs exert more potent cytotoxicity against several human cell lines compared with α-CDs or γ-CDs, and the functional groups on the CDs impact their cytotoxic capacity. The capacity of CDs to interact with cholesterol was shown to be critical for their ability to induce cytotoxicity, and the lipid/cholesterol components in the blood significantly attenuated the hemolytic potency of the methyl β-CDs. Among 12 CD derivatives, HPβCD and HPγCD were shown to reduce the intracellular cholesterol accumulation in NPC patient-derived fibroblasts. We have identified a set of proteins that are either upregulated or downregulated in NPC patient-derived cells upon treatment with HPβCD and HPγCD using a proteomic approach. We propose that selected cellular proteins, altered by HPβCD and/or HPγCD, may play an important function in the regulation of intracellular cholesterol homeostasis. Understanding the molecular mechanisms of HPβCD and HPγCD for rescuing the cholesterol trafficking defect will aid in the design of effective therapeutic approaches for neurodegenerative lipid storage disorders such as NPC disease.

## Figures and Tables

**Table 1:** Cholesterol-solubilizing activity of various cyclodextrin derivatives (50 mM).

	DIMEB-50 ^a^	DIMEB-95 ^b^	RAMEB ^c^	HPβCD ^d^	HPγCD ^e^	SBEβCD ^f^
Cholesterol (mg/mL)	5.68 ± 0.09	4.56 ± 0.04	4.97 ± 0.05	0.75 ± 0.003	0	0.08 ± 0.001

^a^ DIMEB-50: Heptakis (2, 6-di-*O*-methyl)-β-cyclodextrin, isomeric purity 50%; ^b^ DIMEB-95: Heptakis (2, 6-di-*O*-methyl)-β-cyclodextrin, isomeric purity 95%; ^c^ RAMEB: Randomly methylated β-cyclodextrin; ^d^ HPβCD: Hydroxypropyl-β-cyclodextrin; ^e^ HPγCD: Hydroxypropyl-γ-cyclodextrin; ^f^ SBEβCD: Sulfobutyl ether β-cyclodextrin.

**Table 2:** Hemolytic EC_50_ (mg/mL) values of different β-cyclodextrin derivatives on isolated human erythrocytes in PBS in the absence or presence of plasma.

Test System	DIMEB-50 ^a^	DIMEB-95 ^b^	RAMEB ^c^	TRIMEB ^d^
No plasma	1.2 ± 0.2	1.2 ± 0.3	2.3 ± 0.3	7.3 ± 0.2
Plasma 50 μL	8.5 ± 0.2	4.7 ± 0.4	5.2 ± 0.3	11.3 ± 0.3
Plasma 100 μL	9.4 ± 0.4	6.5 ± 0.6	7.0 ± 0.4	14.7 ± 0.4
Plasma 200 μL	11.9 ± 0.3	8.8 ± 0.5	9.7 ± 0.5	19.0 ± 0.2

^a^ DIMEB-50: Heptakis (2, 6-di-*O*-methyl)-β-cyclodextrin, isomeric purity 50%; ^b^ DIMEB-95: Heptakis (2, 6-di-*O*-methyl)-β-cyclodextrin, isomeric purity 95%; ^c^ RAMEB: Randomly methylated β-cyclodextrin; ^d^ TRIMEB: Heptakis (2, 3, 6-tri-*O*-methyl)-β-cyclodextrin.

**Table 3:** Effect of the actual cholesterol levels on the hemolytic EC_50_ (mg/mL) values of DIMEB-50.

Test System	Plasma Added (μL)	CHO ^a^ in Plasma (mM)	HDL ^b^ in Plasma (mM)	TG ^c^ in Plasma (mM)	Hemolytic EC_50_ (mg/mL)
RBCs-washed ^d^	-	-	-	-	1.32 ± 0.1
RBCs-washed ^d^	200	0.76	0.25	0.13	12.6 ± 0.2
Whole blood ^e^	-	2.22	1.13	0.48	18.0 ± 0.2
Whole blood ^e^	-	4.38	0.58	1.08	22.4 ± 0.4

^a^ CHO: cholesterol; ^b^ HDL: high-density lipoprotein; ^c^ TG: triglyceride; ^d^ The red blood cell (RBC) count in the test sample was set at 5 × 10^7^; ^e^ The RBC count in the test sample was set at 1 × 10^9^.
